# Near Infrared Spectroscopy as a Green Technology for the Quality Prediction of Intact Olives

**DOI:** 10.3390/foods10051042

**Published:** 2021-05-11

**Authors:** Silvia Grassi, Olusola Samuel Jolayemi, Valentina Giovenzana, Alessio Tugnolo, Giacomo Squeo, Paola Conte, Alessandra De Bruno, Federica Flamminii, Ernestina Casiraghi, Cristina Alamprese

**Affiliations:** 1 Department of Food, Environmental, and Nutritional Sciences (DeFENS), Università degli Studi di Milano, Via G. Celoria 2, 20133 Milan, Italy; silvia.grassi@unimi.it (S.G.); olusola.jolayemi@unimi.it (O.S.J.); ernestina.casiraghi@unimi.it (E.C.); 2 Department of Agricultural and Environmental Sciences (DiSAA), Università degli Studi di Milano, Via G. Celoria 2, 20133 Milan, Italy; valentina.giovenzana@unimi.it (V.G.); alessio.tugnolo@unimi.it (A.T.); 3 Department of Soil Plant and Food Sciences (DiSSPA), Università degli Studi di Bari “Aldo Moro”, Via Amendola 165/A, 70126 Bari, Italy; giacomo.squeo@uniba.it; 4 Department of Agricultural Sciences, Università degli Studi di Sassari, Viale Italia 39/A, 07100 Sassari, Italy; pconte@uniss.it; 5 Department of Agraria, University Mediterranea of Reggio Calabria, Via dell’Università 25, 89124 Reggio Calabria, Italy; alessandra.debruno@unirc.it; 6 Faculty of Bioscience and Technology for Agriculture, Food and Environment, University of Teramo, Via Balzarini 1, 64100 Teramo, Italy; fflamminii@unite.it

**Keywords:** antioxidant activity, harvesting time, olive composition, olive cultivars, olive ripening, phenolic compounds, PLS regression model, portable device, quality parameters, sustainability

## Abstract

Poorly emphasized aspects for a sustainable olive oil system are chemical analysis replacement and quality design of the final product. In this context, near infrared spectroscopy (NIRS) can play a pivotal role. Thus, this study aims at comparing performances of different NIRS systems for the prediction of moisture, oil content, soluble solids, total phenolic content, and antioxidant activity of intact olive drupes. The results obtained by a Fourier transform (FT)-NIR spectrometer, equipped with both an integrating sphere and a fiber optic probe, and a Vis/NIR handheld device are discussed. Almost all the partial least squares regression models were encouraging in predicting the quality parameters (0.64 < R^2^_pred_ < 0.84), with small and comparable biases (*p* > 0.05). The pair-wise comparison between the standard deviations demonstrated that the FT-NIR models were always similar except for moisture (*p* < 0.05), whereas a slightly lower performance of the Vis/NIR models was assessed. Summarizing, while on-line or in-line applications of the FT-NIR optical probe should be promoted in oil mills in order to quickly classify the drupes for a better quality design of the olive oil, the portable and cheaper Vis/NIR device could be useful for preliminary quality evaluation of olive drupes directly in the field.

## 1. Introduction

The economic significance of olive industries to the European Union is unquestionable. Europe contributed almost 70% of the world olive oil production in the 2018–2019 harvest year campaign and the resultant revenue was to the tune of five billion euro [1]. This large and continuously expanding industry is also associated with many negative environmental problems stemmed from waste production and inappropriate disposal, soil depletion, and atmospheric emissions [2]. Every phase in the olive chain is characterized by different environmental concerns. In the agronomic phase, the use of pesticides, herbicides, and fertilizers has been identified as the principal contributor to ecological challenges [3]. In the cultivation phase, activities such as irrigation, pruning, soil management, and fertilizer applications can negatively affect the environment. The impacts of these primary phases are minor when compared to olive oil production and its unit operations. Oil extraction generates the most potentially hazardous organic compounds that accompany olive wastewater and pomace, depending on the techniques [4]. Laudable efforts have been made to adopt sustainable agricultural and industrial practices in the olive value chain to mitigate these problems. For instance, adoption of organic integrated agricultural systems in the farming and cultivation of olives is an example of sustainable agricultural practice. Industrially, practices such as the two-phase olive extraction method, which reduces water consumption, extraction of bioactive phytonutrients from by-products, and overall valorization of the olive production chain have significantly reduced the negative impacts of the industry on the environment [5,6]. However, a rather less emphasized aspect of the sustainable olive system is solvent reduction and replacement strategies during laboratory chemical analyses of olives and olive oils.

These chemical analyses are fundamental to monitor olive ripeness, estimate oil extraction efficiency, and control oil quality. Free acidity, moisture, and oil contents are examples of chemical parameters serving as quick tests on olive drupes before extraction [7]. On-field information of these chemical parameters can suggest suitable harvest time and overall orchard management [8,9]. Immediate first-hand knowledge of moisture and oil content of olive drupes prior to processing can reliably predict the economic viability of the entire production process, therefore informing producers about the raw material composition is of crucial relevance [10,11]. Similarly, prediction of minor constituents such as phenols, pigments, and antioxidants contents of olives can facilitate instant classification of the resultant oils even before production, making official standard compliance and product consistency easier. Commonly used wet methods, such as Soxhlet extraction technique, gravimetry, and chromatography have many unsustainable limitations such as excess solvent consumption, limited sample size, destructive sample preparation, slow response, and technical demand [7]. Thus, for effective processing and quality control of the olive system, application of green, sustainable eco-friendly, energy-efficient, non-destructive, non-invasive, easy-to-use, and inexpensive spectroscopic methods become inevitable.

From the technological point of view, the importance of these rapid determinations before oil extraction may lie in the possibility of modulating the extraction systems based on the drupe characteristics and type of desired product. For instance, operative conditions safeguarding the phenolic content can be adopted if phenolic substances are not so high in the drupes or, vice versa, the outstanding phenolic content of some drupes can be lowered if the final product is intended for consumers who do not like bitter/pungent oil [12,13]. Knowing how to set the equipment before starting the process instead of correcting the settings once the oil has been extracted and analyzed might be of interest.

Near infrared spectroscopy (NIRS) has gained prominence in the last decade and has contributed economically to food and feed industries by ensuring on-time processing and quality control [14,15]. The technology is a formidable green chemistry tool and environmentally sustainable analytical technique capable of handling a large sample size in solid and liquid forms and it provides quick answers to quality questions. NIRS, in conjunction with appropriate chemometrics, has become a routine analytical tool for the determination of intact olive drupes moisture and fat contents [16,17]. Using a portable Vis/NIR spectral acquisition device equipped with multiple detectors, it was possible to predict several economically important olive mill parameters such as maturity index, moisture, oil content, acidity, and dry matter [18]. Another type of NIRS system with a wavelength selection tool (acousto-optically tunable filter—AOTF) was satisfactorily applied to predict phenolic compounds and to monitor ripening of olives [19,20]. In addition to intact or crushed olive quality assessment, NIRS has been found to be handy in evaluation of olive oils and olive by-products [21,22]. However, comparative performance evaluations of NIRS using different signal acquisition devices are relatively uncommon especially for olive drupes. In this study the results obtained by a Fourier transform (FT)-NIR spectrometer (equipped with both integrating sphere and fiber optic probe) and a Vis/NIR handheld device for the prediction of quality parameters of intact olives of 13 different cultivars collected in three harvest years are discussed. In particular, the objective was to evaluate the different performance of the acquisition systems in the prediction of moisture, oil content, soluble solids, total phenol content, and antioxidant activity, in vision of suitable tools to be applied both in the field and at the mill for quick answers to quality questions in a sustainable way.

## 2. Materials and Methods 

### 2.1. Olive Samples

Samples of olives belonging to 13 different cultivars from Abruzzo, Apulia, Calabria, and Sardinia regions (Italy) were used; sampling was carried out at different ripening degrees during 2016–2018 harvesting years. For each sampling and cultivar, three sample units (500 g each) were picked from different identified trees of the same grove, for a total of 267 sample units. Each unit was independently analyzed for the chemical parameters (moisture, oil content, soluble solid content, total phenolic content, and antioxidant activity). Two aliquots (100 g each) were taken from each sample unit for FT-NIR analysis with the integrating sphere. From each aliquot, 10 olives were selected as representative of the ripening stage [23] and used for analyses with both the FT-NIR and Vis/NIR fiber optical probes.

### 2.2. Chemical Analyses

Determination of moisture content (%) was carried out according to the AOAC 934.06 official method [24]. Oil content (% on fresh weight) was determined gravimetrically after the extraction of the oil from 10 g of dehydrated olive paste in a Soxhlet apparatus using petroleum ether as solvent [25]. Total soluble solids content (°Bx) was measured according to a previously published procedure [26]. Briefly, the sugar aqueous solution was prepared by homogenizing olive paste (20 g) in distilled water (40 mL) and stirring for 2 min. After centrifugation (11,000× *g* for 10 min), the supernatant solution was analyzed through a digital refractometer. Total phenol content (TPC) was determined as follows: olive pulp (1 g) was extracted using hexane (3 mL) and methanol:water (70:30 *v*/*v*; 15 mL), by stirring for 10 min at room temperature. After centrifugation (6000× *g* at 4 °C for 10 min), the supernatant phase was collected and further centrifuged (13,600× *g*, 5 min, room temperature). The obtained extracts were filtered through nylon syringe filters (pore size 0.45 μm; LLG Syringe Filter CA, Carlo Erba, Milano, Italy), properly diluted, and spectrophotometrically analyzed at 750 nm using the Folin-Ciocalteau reagent [27]. Calibration curves were made using gallic acid and the results were expressed as grams of gallic acid equivalent per kilogram olive pulp (g_GA_/kg). Antioxidant activity (% inhibition/mg olive pulp) was determined on the same extracts used for TPC, applying the radical 2,2 diphenyl-1-picrylhydrazyl (DPPH^•^) method [28]. Briefly, 200 μL extract (previously diluted 1:20 in methanol) was made to react with 2.8 mL DPPH^•^ methanol solution (6 × 10^−5^ M) for 1 h at 22 °C, measuring the discoloration at 515 nm. All reagents were from Sigma-Aldrich (St. Louis, MO, USA). 

### 2.3. Spectra Collection

Spectra were collected by using a benchtop FT-NIR spectrometer (MPA, Bruker Optics, Milan, Italy), equipped with both an integrating sphere and a fiber-optic probe, and a handheld portable Vis/NIR device (Jaz, OceanOptics Inc., Dunedin, FL, USA). The FT-NIR spectra of the two aliquots (100 g each) of each olive sample unit were collected in duplicate in diffuse reflectance by means of the integrating sphere system. The optical fiber was used to acquire, in duplicate, the FT-NIR spectra of the 10 single olives selected from each aliquot based on ripening degree [23]. For both FT-NIR sampling systems, spectra were collected within a 12,500–3600 cm^−1^ spectral range, at 8 cm^−1^ resolution and with 32 scans. The background for the integrating sphere was performed by closing the internal reference wheel of the module, while for the fiber-optic probe a Spectralon standard was used. A dedicated software (OPUS v. 6.5, Bruker Optics, Ettlingen, Germany) was used to manage the instrument. The same single olives were analyzed in duplicate also by using the Vis/NIR portable device (500–1000 nm, i.e., 20,000–10,000 cm^−1^; 0.3 nm resolution; 5 scans) equipped with a bifurcated optical fiber provided with a cap that standardizes the distance between the head of the probe and the sample (about 2 mm) and reduces the environmental light interference. A white reference (99% reflection) was used to set the maximum reflection. Spectrum acquisition lasted 18 s for both the integrating sphere and the probe of the benchtop FT-NIR spectrometer, and 1 s for the portable Vis/NIR device. Measurements were conducted with both instruments on the same day, thus making sample storage between analyses unnecessary.

### 2.4. Data Analysis

Data elaborations were performed using the Unscrambler X software (v. 10.4, CAMO ASA, Oslo, Norway). The replicated spectra were averaged in order to have one spectrum for each sample unit. For FT-NIR probe and sphere, spectral ranges were reduced to eliminate non-informative and noisy regions (i.e., 3600–4000 and 10,500–12,500 cm^−1^), whereas in the case of the portable Vis/NIR device, the whole spectral range was used. The spectral data were independently pre-processed by standard normal variate (SNV), which removes possible interferences due to light scattering [29]. Chemical variables and all spectral data were merged in a single matrix (267 sample units × 5024 variables) and used to perform principal component analysis (PCA), autoscaling all the variables to overcome the heteroscedasticity nature of the data. The coordinate transformation of the merged spectral–chemical data matrix allowed for the selection of a calibration and a prediction data set, using the Kennard–Stone (KS) algorithm [30]. The algorithm partitioned the data in order to have 70% of samples (187 sample units) in the calibration set and 30% (80 sample units) in the prediction set.

Prediction of olive chemical characteristics based on spectral data was performed applying the partial least squares (PLS) regression to the calibration set of each spectral matrix (187 sample units × 1686 variables for the FT-NIR systems; 187 sample units × 1647 variables for the Vis/NIR equipment) using nonlinear iterative partial least squares (NIPALS) algorithm. Different pre-treatments of spectral data were tested: SNV, first derivative (d1; Savitzky–Golay algorithm, second order polynomial, 11-window size), which allows removal of baseline offset [31], and their combination. After calibration, the models were validated internally, through cross-validation (Venetian blind, 10 cancellation segments). The number of components to be considered for each model was determined based on the plot of calibration and cross-validation errors as a function of the number of latent variables (LVs). The optimal number of LVs was chosen as the number of LV allowing to minimize the cross-validation error. Afterwards, the models were externally validated by independently using the prediction set previously created with KS. Model performance was evaluated in terms of determination coefficients for calibration (R^2^_cal_), cross-validation (R^2^_cv_), and prediction (R^2^_pred_), as well as by root mean square error of calibration (RMSEC), cross-validation (RMSECV), and prediction (RMSEP), and standard error of prediction (SEP).

Prediction performances of the models obtained by the three spectral acquisition systems were compared by different approaches: (i) comparison of intermediate precisions expressed as standard error of laboratory (SEL); (ii) comparison of SEP with SEL of reference analyses; (iii) statistical tests proposed in the scientific literature [32,33]. SEL of the reference analyses and NIRS acquisition systems was calculated as follows [34]:

$$SEL=\sqrt{\frac{\sum_{1}^{m}{\left(x_{1}-x_{2}\right)}^{2}}{m}}$$

where *m* is the number of olive samples and *x_1_ − x_2_* is the absolute value of the difference between replicate results. In the third approach (i.e., statistical tests), first, the model biases, i.e., differences between the reference method results and those of the models predicting the chemical parameters, were compared by a *t* confidence interval for paired samples with a 95% confidence interval. The null hypothesis (H_0_) states that model biases are not different. If the calculated Fisher value is higher than the F critical value, the H_0_ is rejected and the hypothesis H_1_ is true (i.e., differences between models are significant) [32]. Furthermore, a pairwise comparison of the model standard deviations was performed by the calculation of the correlation coefficient between each two sets of prediction errors (r). Then, K index is calculated by the following equation:

K = 1 + {[2(1 − r^2^)t^2^_n−2,0.025_]/(n − 2)} (1)

where t_n−2,0.025_ is the upper 2.5% point of the *t* distribution on n − 2 degrees of freedom. Subsequentially, L index is calculated as follows [33]:

L = √[K + √((K^2^ − 1))] (2)

Then, the 95% confidence interval for the ratio of the standard deviations (L-lower and L-upper limits) was calculated. If the L interval includes 1, the standard deviations are not significantly different (*p* > 0.05). The model comparison was performed in MATLAB environment (v. R2017b, The MathWorks, Inc., Natick, MA, USA).

## 3. Results and Discussion

### 3.1. Chemical Parameters

**Figure 1:**
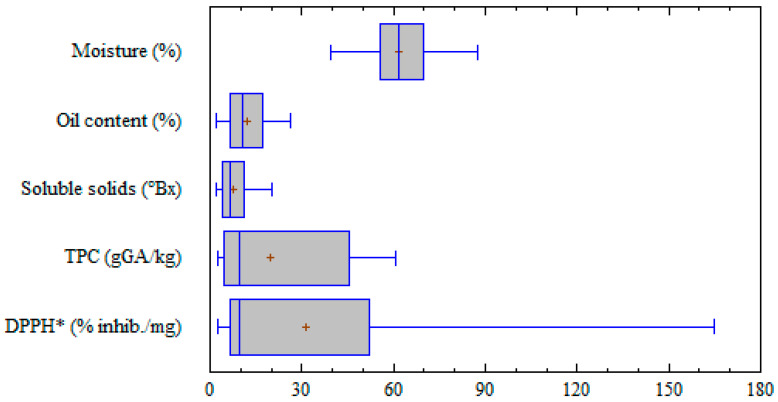
Box and whisker plots showing the descriptive statistics for the chemical variables tested on olive drupes. TPC: total phenol content; GA: gallic acid equivalent; DPPH^•^: radical 2,2 diphenyl-1-picrylhydrazyl; inhib.: inhibition.

Descriptive statistics of the chemical variables are presented in Figure 1 as box and whisker plots. The box lines represent the first and third quartiles and the median. The mean value is indicated by a cross sign. Whiskers correspond to the minimum and maximum measured values. Genetic, environmental, and cultivation factors affect olive composition, which changes during growth together with the drupe weight [5]. Actually, the tested cultivars and the different ripening stages and crop seasons accounted for a high range of variability of all the chemical parameters. This is an important point for the development of prediction models useful for different production sites. Variation ranges of the chemical parameters for the different olive cultivars are reported in Table S1.

Moisture represents the main constituent alongside oil. In the considered drupes, moisture content ranged from 39.3 to 87.2%. The obtained results agree with previously published data [18,35], considering that the moisture mean value was 63.3%, while the highest values (>80%) were obtained only in three out of thirteen cultivars, all from Calabria region. Excluding those three cultivars, the maximum value for moisture was 73.7%.

Commonly, olives intended for oil production have approximately 20% oil [36]. The samples here considered had a wide range of oil content (1.9–26.0%), suggesting the high influence of cultivar and ripening degree on this parameter. A general increase in oil content ranging from 2 to 12% was observed over ripening, depending on the considered cultivar.

TPC is an approximate estimation of total phenolic acids, phenolic alcohols, flavonoids, and secoiridoids in olive drupes. These compounds confer the bitter taste and pungent sensation on olive oils and are responsible for the well-known antioxidant properties. TPC values of the samples had a wide range of variation (2.5–60.6 g_GA_/kg), with the highest levels (>35 g_GA_/kg) found in three cultivars from Sardinia region. The antioxidant activity too was very different in the various samples, ranging from 2.4 to 165.0% inhibition/mg. Unexpectedly, the highest values (>70% inhibition/mg) were not found in the olives with the highest TPC, but in two cultivars from the Apulia region. 

### 3.2. Spectral Features

**Figure 2:**
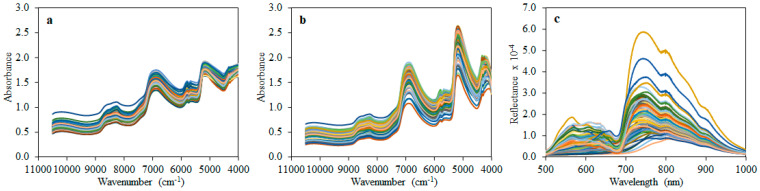
Spectra of olive drupes acquired with: (**a**) FT-NIR integrating sphere; (**b**) FT-NIR fiber-optic probe; (**c**) portable Vis/NIR device.

Figure 2 shows the spectra of the olives obtained from the three acquisition systems. Visual features and patterns of the spectra conform with those previously reported for intact olive drupes [37,38].

Aside from the visual differences in band intensities among samples, FT-NIR spectra from the integrating sphere and the fiber-optic probe (Figure 2a,b) are quite similar, with the latter exhibiting higher absorbances in most of the observable peaks.

The low absorbance band around 8600 cm^−1^ represents a combined symmetric and asymmetric OH stretching and bending vibrations. This is followed by the second overtone of CH stretching vibrations at 8300 cm^−1^ that corresponds to methyl (-CH_3_), methylene (-CH_2_), and olefin (-CH=CH-) bonds [37]. The high water content of the olive drupes (39–87%) explains the two absorption bands at 7500–6100 and 5400–4500 cm^−1^. These bands are designated as the combination of first overtone of symmetric and asymmetric OH-bending and OH-stretching bands (6900 cm^−1^) and combined OH-bending and OH-stretching bands (5200 cm^−1^), respectively [39]. Similarly to the second overtone of CH stretching vibrations at 8300 cm^−1^, the two bands at 5800 and 5650 cm^−1^ represent the first overtone of CH-stretching vibrations present in the same CH_3_, CH_2_, and CH=CH functional groups. At the far end of the FT-NIR spectral range, two peaks at 4335 and 4262 cm^−1^ represent CH and CH_2_ s overtones, respectively [35]. However, the intermediate bands between the overtones (i.e., 8600, 5800–5650, and 4350–4250 cm^−1^) have been attributed to the oil content of the drupes [40]. Regarding olive fruit phenols, there are no reported NIR correlated bands in the literature. However, a previous study suggested that some regions (i.e., 8700–8300 and 5800–5650 cm^−1^) are correlated with TPC of olives [19].

In the case of Vis/NIR spectra (Figure 2c), more peak variations among samples were observed, especially within the visible (550–680 nm) and near-infrared (700–790 nm) regions. The changes around 550–680 nm correspond to some varying pigment indices. Specifically, the peak around 540 nm has been associated with anthocyanin, while that at 680 nm has been linked to chlorophyll [41]. Thus, changes in reflectance along these peaks may be due to maturation differences among the drupes. Other parameters, such as soluble solids, pH, and firmness, have been implicated within these regions in pears, especially around 340–740 nm [42]. Changes in the two absorption peaks around 750 and 850 nm could be assigned to the third overtone of H_2_O and C-H functional group, respectively [43]. 

### 3.3. Principal Component Analysis

**Figure 3:**
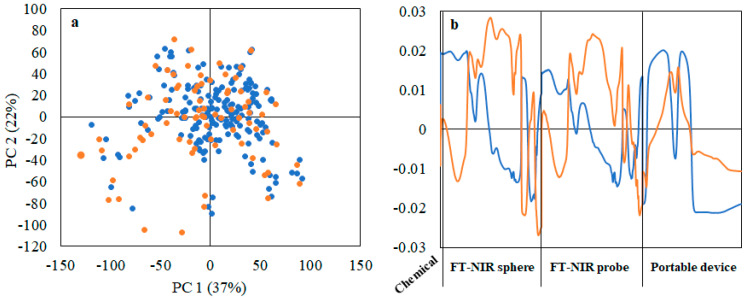
PCA results: (**a**) score plot showing the distribution of calibration (blue) and prediction (orange) set samples selected by Kennard-Stone algorithm applied on the merged chemical and spectral dataset of olive drupes; (**b**) loading plot of PC1 (blue) and PC2 (orange).

Figure 3 shows the score and loading plots of the PCA model built on the merged chemical and spectral database. The first two principal components (PCs) represent 59% of total data variance. The application of KS algorithm after PCA allowed to select evenly distributed samples for the calibration and prediction sets, highlighted with different colors in the score plot of Figure 3a. Few samples were seemingly outliers, but they were not removed in order to avoid presumptive assumption that they might adversely affect the model. Anyway, KS data splitting algorithm retained to a large extent as much variability as possible within the calibration and validation sets and this is a prerequisite for model robustness and validity in prediction. The loading plot (Figure 3b) shows a balanced contribution of both the chemical parameters and the three spectral ranges to sample distribution and consequently to the dataset partitioning.

### 3.4. Regression Models

PLS regression models were built with FT-NIR and Vis/NIR spectra to quantify moisture, oil content, soluble solids, TPC, and antioxidant activity of olive drupes. In order to make data more evenly distributed, TPC and DPPH^•^ results were transformed in the inverse and the logarithmic values, respectively. The best models based on determination coefficients and errors are reported in Table 1 for each spectral acquisition system. Predicted vs. measured plots of the models are reported in the Supplementary Figure S1. In general, performances of the three acquisition systems were similar in calibration and cross-validation, while in prediction FT-NIR spectra gave better results, maybe due to the wider NIR range and the low complexity of the models resulting in a higher stability.

With respect to moisture content prediction, the three acquisition systems exhibited promising and similar prediction outcomes. The determination coefficients ranged from 0.77 to 0.92, with reasonably low values of errors (from 2.67 to 4.75%). However, the model calculated with the Vis/NIR spectra transformed in d1 showed a higher number of LVs (16 vs. 8 and 7 for the sphere and the probe, respectively), maybe due to the higher resolution of the spectra and the limited NIR range considered.

Oil content was better predicted by FT-NIR spectra, pre-treated with SNV and d1. Prediction coefficients of determination were higher than those of the portable acquisition system (0.77 and 0.78 vs. 0.64), with lower RMSEP values (2.92 and 2.86% vs. 3.74%) and LVs (9 and 5 vs. 16). The outcomes of calibration and cross-validation coefficients of determination for the FT-NIR sphere and probe (0.77–0.93) were comparable to those reported in the literature (0.78–0.84) for a smaller number of samples (183) [18].

Considering soluble solids, the regression model reliability appeared even more promising for FT-NIR spectrometer than for the portable device. Both the FT-NIR sphere and fiber-optic spectra pre-treated with a combination of SNV and d1 resulted in satisfactory determination coefficients in prediction (0.70 and 0.74, respectively) and low RMSEP (2.39 and 2.23 °Bx, respectively). The precision of the models was comparable to those observed for other fruits as, to the best of our knowledge, there is no study on NIR prediction of soluble solids in intact olives. For instance, quantitative determination of soluble solid content for quality prediction of intact strawberries using a handheld micro-electro-mechanical NIR system, resulted in R^2^_pred_ of 0.37–0.47 and RMSEP of 1.02–0.87% [44]. With the spectra in the Vis/NIR range, the coefficient of determination in prediction decreased to 0.58, with a RMSEP of 3.02 °Bx.

Similar model performances in calibration and cross-validation were obtained for 1/TPC for all the spectral acquisition systems (R^2^ range, 0.76–0.89), whereas in prediction FT-NIR spectra, gave better results (R^2^_pred_ = 0.77–0.76) than Vis/NIR spectra (R^2^_pred_ = 0.69). FT-NIR models are better than those reported in the literature for a filter-based NIR spectrometer [35]. The authors attributed the unsatisfactory output of their model (R^2^_cal_, 0.72; SEP, 13.35 g oleuropein/kg_dm_) to the exclusion of 8600–6900 cm^−1^ range from the spectral bands, which was instead here considered. Our models were more promising also when compared to grape TPC prediction models developed using a portable NIR-AOTF [45]; the authors observed determination coefficient values of 0.77 and 0.62 in calibration and cross-validation, respectively.

To the best of our knowledge, there is no other published paper in which the antioxidant activity of olive drupes is tentatively determined using rapid spectroscopic techniques. Therefore, our models seem fair especially when the FT-NIR probe was used, which generated comparatively highest R^2^_pred_ and lowest RMSEP among the three spectral acquisition systems. The dynamic nature of this in vitro antioxidant activity makes its adaptation to spectroscopic techniques somewhat difficult. A more accurate NIR prediction of DPPH^•^ radical scavenging activity was recorded in bean flours (R^2^_cal_, 0.94–0.99; R^2^_val_, 0.85–0.97) [46]. On the contrary, for a more bioactive horticultural product like *Hibiscus sabdariffa*, calibration and prediction determination coefficients are reported in the literature in the ranges 0.82–0.87 and 0.75–0.86, respectively, depending on spectra pretreatments [47].

From the inspection of the weighted regression coefficients of PLS models, for both the FT-NIR sphere and the probe the relevance of 7500–6100 and 5400–4500 cm^−1^ regions for moisture and soluble solid prediction was confirmed. Moreover, the PLS model developed for oil content prediction were highly influenced by the 5800–5650 and 4350–4250 cm^−1^ regions, attributed to the oil content of the drupes [40]. The same regions showed high weighted regression coefficients for TPC and DPPH^•^ models, which were also characterized by high relevance of the 8700–8300 cm^−1^ region, previously related to TPC of olives [19].

As for the model developed with the Vis/NIR spectra, the inspection of the weighted regression coefficients revealed that both visible and NIR range influenced the prediction of moisture, oil content, and soluble solids. In particular, the range 880–970 nm showed the highest influence in the models for moisture and oil content prediction, whereas the maximum recorded weight for soluble solids corresponded to 970 nm. Moving to TPC and DPPH^•^ prediction, it has been noticed that the highest values of the weighted regression coefficients were related to the visible range (550–700 nm), maybe linked to the olive color modification occurring during ripening, due to compounds like chlorophylls, carotenoids, anthocyanins, and polyphenols. Actually, other authors demonstrated that during olive ripening a rise in some bands of the visible range occurs (i.e., 600–650 and 550–625 nm), due to the presence of anthocyanin and other pigments related to reddish as well as green and yellow color [18].

### 3.5. Regression Model Comparison

The effectiveness of the prediction ability was at first established comparing the intermediate precisions (SEL) of the regression models with those of the reference methods (Table 2). The SEL values for the different NIR systems were generally higher than those obtained for the reference analyses, except for the SEL of the oil content predicted by the FT-NIR probe measurements. Indeed, the SEL values of NIR systems are more affected by the drupe heterogeneity, since spectra are collected on entire olives without the sample preparation phase of the chemical analyses, which is carried out by grinding and homogenizing the olive pulp.

The SEL_ref_ values were also compared with the prediction performances of the models in terms of SEP. As expected, SEP values were always higher than those of SEL_ref_, because they include not only the sampling and analysis errors, but also the spectroscopy and model errors. The SEP obtained for the FT-NIR probe models were the lowest and the closest to the corresponding SEL_ref_ values. If the SEP is <2SEL_ref_, the prediction performance of the model should be considered as good [48]. This was the case of models developed from FT-NIR probe spectra for moisture, oil content, and 1/TPC prediction. 

Furthermore, the *t*-test for paired samples demonstrated that the biases for the models developed with the three spectral acquisition systems were comparable, i.e., the null hypothesis was confirmed (*p* values between 0.1 and 0.8; data not shown). On the other hand, the comparison between the standard deviations of the models [33] returned some differences as reported in the last three columns of Table 2. For moisture, the FT-NIR probe model resulted significantly different from those based on sphere and portable device spectra, due to a better performance resulting in a lower RMSEP (Table 1). All the other comparisons resulted in similar performance of the FT-NIR sphere and probe models, whereas the portable device models resulted significantly different because of the worse performance in terms of R^2^_pred_, RMSEP, and SEP.

## 4. Conclusions

The benefits of different NIRS acquisition systems as green technology for quality characterization of intact olive drupes were explored. Generally, the calculated PLS models were remarkably encouraging in terms of determination coefficients and errors, both in internal validation and prediction. The model comparison highlighted a general better performance of both the FT-NIR sphere and probe acquisition systems with respect to the handheld device. However, the Vis/NIR device, being portable and relatively cheaper, is worthy of further investigations, because its use could be in any case very useful for preliminary quick quality assessment of olive drupes directly in the field. On the contrary, an on-line or in-line application of the FT-NIR optical probe in the olive mill should be promoted in order to quickly classify the drupes for a better quality design of the olive oil and a more sustainable management of the production chain.

## Figures and Tables

**Table 1:** Figures of merit of the best PLS regression models for olive chemical parameter prediction based on spectroscopic data.

				Calibration		Cross-Validation		Prediction	
Parameter	NIR System	Pre-treatment	LVs	R^2^_cal_	RMSEC	R^2^_cv_	RMSECV	R^2^_pred_	RMSEP
Moisture content (%)	Sphere	SNV+d1	8	0.92	2.67	0.85	3.66	0.77	4.59
	Probe	SNV+d1	7	0.88	3.56	0.85	3.87	0.84	3.97
	Portable	d1	16	0.87	3.68	0.77	4.77	0.77	4.75
Oil content (%)	Sphere	SNV+d1	9	0.93	1.62	0.82	2.62	0.77	2.92
	Probe	SNV+d1	5	0.79	2.87	0.77	2.99	0.78	2.86
	Portable	SNV+d1	16	0.81	2.72	0.67	3.58	0.64	3.74
Soluble solids (°Bx)	Sphere	SNV+d1	9	0.90	1.45	0.75	2.36	0.70	2.39
	Probe	SNV+d1	11	0.87	1.66	0.80	2.06	0.74	2.23
	Portable	SNV	13	0.79	2.11	0.75	2.34	0.58	3.02
1/TPC (kg/g_GA_)	Sphere	SNV	13	0.89	0.04	0.81	0.04	0.77	0.04
	Probe	SNV+d1	13	0.87	0.04	0.76	0.05	0.76	0.04
	Portable	SNV+d1	9	0.83	0.05	0.79	0.05	0.69	0.05
logDPPH^•^ (log % inhib./mg)	Sphere	SNV	15	0.84	0.20	0.68	0.29	0.68	0.29
	Probe	SNV+d1	16	0.93	0.14	0.79	0.24	0.73	0.27
	Portable	d1	13	0.79	0.23	0.72	0.27	0.41	0.39

TPC: total phenolic content; GA: gallic acid equivalent; inhib.: inhibition; DPPH^•^: radical 2,2 diphenyl-1-picrylhydrazyl; LVs: latent variables; R^2^_cal_: calibration coefficient of determination; R^2^_cv_: cross-validation coefficient of determination; R^2^_pred_: prediction coefficient of determination; RMSEC, RMSECV, and RMSEP: root mean square errors of calibration, cross-validation, and prediction, respectively; SNV: standard normal variate; d1: first derivative.

**Table 2:** Comparison of regression models calculated for olive chemical parameter prediction based on three different FT-NIR and Vis-NIR acquisition systems.

Parameter	SEL_ref_	NIR System	SEL_NIR_	SEP	NIR System		
					Sphere	Probe	Portable Device
Moisture content (%)	2.00	Sphere	4.41	4.56	-	*	n.s.
		Probe	3.21	3.99	*	-	*
		Portable	4.49	4.72	n.s.	*	-
Oil content (%)	2.29	Sphere	3.13	2.94	-	n.s.	*
		Probe	2.18	2.88	n.s.	-	*
		Portable	2.95	3.77	*	*	-
Soluble solids (°Bx)	1.02	Sphere	2.21	2.41	-	n.s.	*
		Probe	2.31	2.24	n.s.	-	*
		Portable	1.88	3.03	*	*	-
1/TPC (kg/gGAE)	0.023	Sphere	0.045	0.044	-	n.s.	*
		Probe	0.044	0.043	n.s.	-	*
		Portable	0.036	0.052	*	*	-
logDPPH• (log % inhib./mg)	0.106	Sphere	0.257	0.287	-	n.s.	*
		Probe	0.282	0.267	n.s.	-	*
		Portable	0.223	0.390	*	*	-

TPC: total phenolic content; GA: gallic acid equivalent; DPPH^•^: radical 2,2 diphenyl-1-picrylhydrazyl; inhib.: inhibition; SEL_ref_: standard error of laboratory for reference analyses; SEL_NIR_: standard error of laboratory for NIR systems; SEP: standard error of prediction; n.s.: not significantly different standard deviation values (*p* > 0.05); *: statistically different standard deviation values (*p* ≤ 0.05).

## Supplementary Materials

The following are available online at https://www.mdpi.com/article/10.3390/foods10051042/s1, Figure S1: Regression lines obtained for the prediction of entire olive chemical parameters with models developed by FT-NIR integrating sphere, FT-NIR fiber-optic probe, and portable Vis/NIR device. Table S1: Variation ranges of the chemical parameters for the different olive cultivars.
