# Supplementary material for: Near Infrared Spectroscopy as a Green Technology for the Quality Prediction of Intact Olives
Source: Foods. 2021 May 11;10(5):1042. doi: 10.3390/foods10051042 (PMC8151771; doi:10.3390/foods10051042)
Supplement: Supplementary file 1 [file foods-10-01042-s001.zip › foods-1204253-supplementary.pdf]

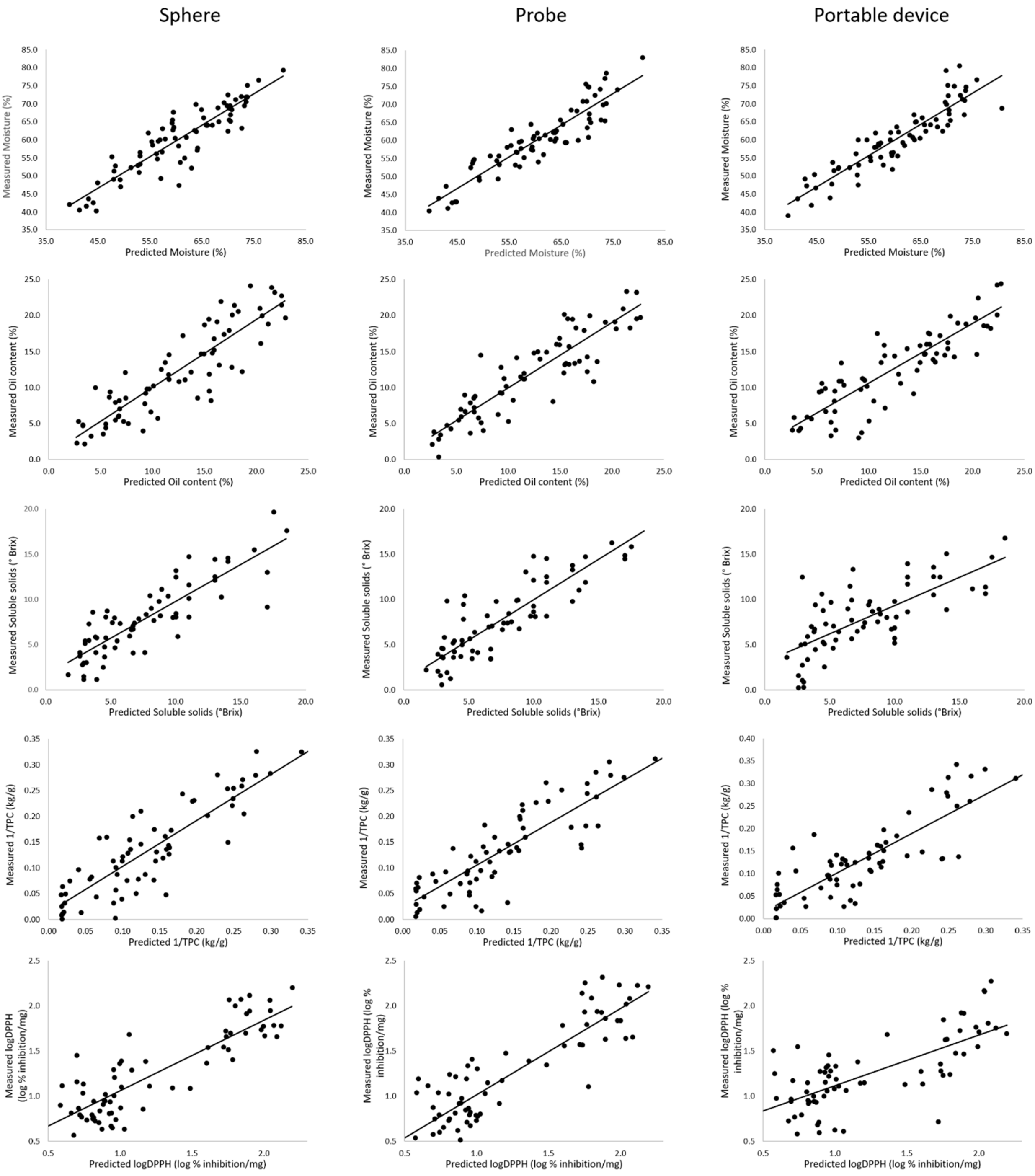

Figure S1. Regression lines obtained for the prediction of entire olive chemical parameters with models developed by FT-NIR integrating sphere, FT-NIR fiber-optic probe, and portable Vis/NIR device.

Table S1. Variation ranges of the chemical parameters for the different olive cultivars.

Region	CV	Moisture content (%)	Oil content (%)	Soluble solids (°Bx)	TPC (g_{GA}/kg)	DPPH (% inhib./mg)
Abruzzo	DR	64.96–73.66	11.55–22.44	9.75–12.2	4.15–10.05	37.38–62.83
Abruzzo	GE	60.77–73.72	9.62–24.12	6.75–170	2.56–13.38	21.50–74.12
Abruzzo	TO	57.86–66.33	13.20–25.97	11.00–15.00	12.02–25.50	73.88–117.56
Apulia	CM	45.84–56.54	14.65–24.19	10.50–17.50	3.77–11.06	78.85–134.78
Apulia	OR	43.11–58.36	16.16–22.58	8.00–19.00	5.55–18.03	94.9–165.07
Apulia	BM	43.71–50.66	19.37–22.49	17.00–20.00	6.05–6.92	58.01–78.66
Calabria	CI	62.28–85.26	3.97–18.70	2.10–5.80	2.48–19.72	4.41–18.62
Calabria	CL	57.52–81.58	4.88–16.17	2.10–3.90	2.47–10.34	2.42–11.59
Calabria	CN	52.84–87.21	1.85–10.43	1.70–8.40	2.93–16.45	4.18–16.48
Calabria	TF	57.02–68.36	6.70–21.00	3.00–5.80	2.89–12.85	3.19–11.40
Sardinia	CO	56.04–65.91	3.85–9.61	5.19–8.74	48.96–60.62	5.45–10.63
Sardinia	SE	52.57–64.02	5.92–11.53	5.27–10.38	46.77–60.02	6.41–10.13
Sardinia	SI	39.29–63.08	7.42–23.50	3.83–8.88	35.57–55.32	4.54–9.10

CV: olive cultivar; TPC: total phenolic content; GA: gallic acid equivalent; inhib.: inhibition; DR: Dritta; GE: Gentile dell'Aquila; TO: Tortiglione; CM: Cima di Malfi; OR: Oliva Rossa; BM: Bambina; CI: Ciciariello; CL: Ottobratica Calipa; CN: Ottobratica Cannavà; TF: Tonda di Filogaso; CO: Corsicana; SE: Semidana; SI: Sivigliana.
